# Potential protective effect of dental treatment among subgroups of critically ill ventilated patients: a retrospective survival analysis

**DOI:** 10.62675/2965-2774.20260305

**Published:** 2026-05-08

**Authors:** Flávio de Melo Garcia, Caroline Tianeze de Castro, Renan Vicente Starling Braga, Josiane Celis de Almeida, Victor Angelo Martins Montalli

**Affiliations:** 1 Universidade de São Paulo Faculdade de Saúde Pública São Paulo SP Brazil Faculdade de Saúde Pública, Universidade de São Paulo - São Paulo (SP), Brazil.; 2 Universidade Federal da Bahia Institute of Collective Health Salvador BA Brazil Institute of Collective Health, Universidade Federal da Bahia - Salvador (BA), Brazil.; 3 Hospital Santa Casa de Poços de Caldas Poços de Caldas MG Brazil Hospital Santa Casa de Poços de Caldas - Poços de Caldas (MG), Brazil.; 4 Faculdade de Odontologia São Leopoldo Mandic Campinas SP Brazil Faculdade de Odontologia São Leopoldo Mandic - Campinas (SP), Brazil.

## INTRODUCTION

Critically ill patients undergoing mechanical ventilation (MV) are at heightened risk for ventilator-associated pneumonia (VAP), with oral care recognized as a key preventive measure.^(1–4)^ We hypothesized that poor oral health could influence VAP risk and that dental interventions may offer additional protective benefits beyond routine hygiene.

## METHODS

**Figure 1:**
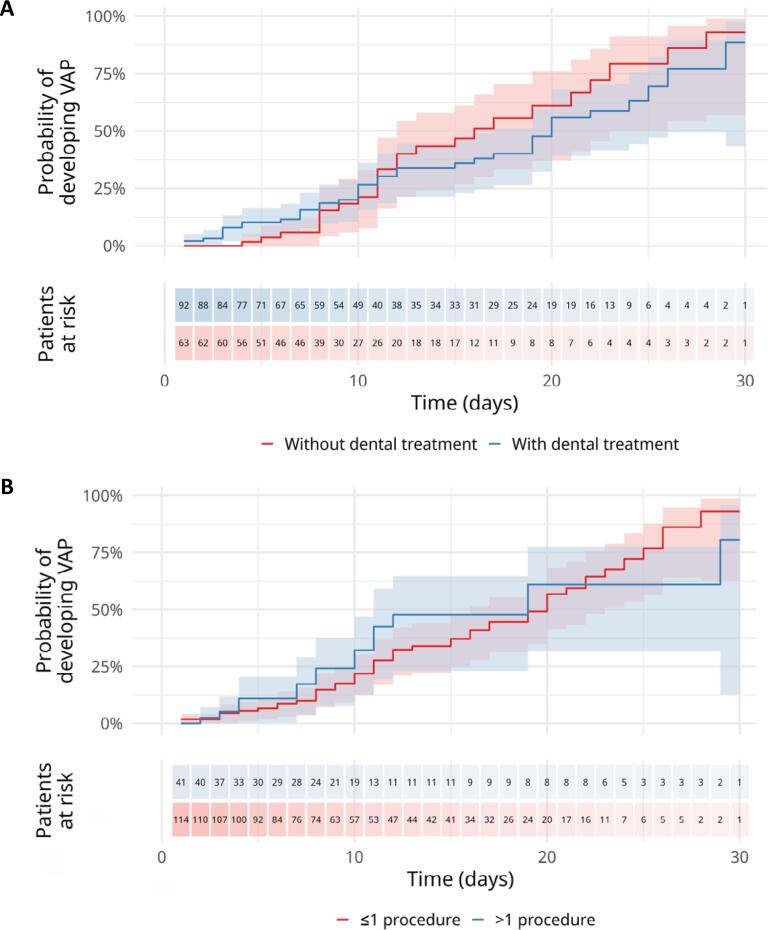
(A) Estimated probability of developing ventilator-associated pneumonia and 95% confidence interval over the duration of mechanical ventilation. According to dental treatment administration (n = 166); (B) estimated probability of developing ventilator-associated pneumonia and 95% confidence interval over the duration of mechanical ventilation. According to the number of dental procedures performed (n = 166).

This observational, longitudinal, and retrospective study followed the STROBE guidelines and was approved by the Ethics Committee of the School of Public Health, *Universidade de São Paulo* (CAEE: 59596422.6.0000.5421).^(5)^ We analyzed secondary clinical and oral health data from medical and dental records of adult patients admitted to the intensive unit care (ICU) of *Hospital Santa Casa de Poços de Caldas*, Minas Gerais, Brazil, between January 1st and August 31st, 2022. The convenience sample included all patients aged ≥18 years who were on MV at ICU admission or initiated MV during their stay (Figure 1S - Supplementary Material). Ventilator-associated pneumonia was diagnosed according to the national guidelines for healthcare-associated infections.^(6)^ Patients were not selected based on oral conditions, allowing for a real-world assessment of ICU dental needs. Definitions of dental treatment and details of statistical analysis are available in tables 1S and 2S (Supplementary Material).

## RESULTS

Among the 166 mechanically ventilated ICU patients with complete temporal data, 39.2% developed VAP. Interaction analyses showed markedly higher VAP risk among patients with prolonged ventilation (> 14 days) without dental treatment (adjusted relative risk [RR] = 21.49; 95% confidence interval [95%CI] 3.03 - 152.30; p = 0.0021) and among those with treatment (adjusted RR = 18.13; 95%CI 2.56 - 128.56; p = 0.0037). Similarly, among patients ventilated > 14 days, VAP risk was high for both ≤ 1 dental procedure (adjusted RR = 6.32; 95%CI 2.67 - 14.94; p < 0.0001) and > 1 procedure (adjusted RR = 6.33; 95%CI 2.55 - 15.75; p < 0.0001) (Table 1). Ventilator-associated pneumonia probability increased with ventilation duration across all groups (e.g., day 15: 0.359 - 0.467; day 30: 0.804 - 0.931). Cox regression showed no significant effect of dental treatment (HR = 1.20; 95%CI 0.72 - 2.00; p = 0.48) or number of dental procedures (≤ 1 *versus* > 1: HR = 1.00; 95%CI 0.56 - 1.77; p = 0.99) on VAP risk (Tables 3S and 4S, Figures 2S, 3S and 4S [Supplementary Material], and Figure 1). Regarding oral health, tongue coating was observed in 36.7% of patients, periodontal disease in 46.7%, edentulism in 14.5%, untreated dental lesions in 16.9%, and oral mucosal lesions in 24.1%. Common dental treatments included photobiomodulation (26.5%), scaling (27.7%), tooth extraction (18.7%), and restorative treatment (6.6%) (Table 5S - Supplementary Material).

**Table 1:** Risk analysis of ventilator-associated pneumonia in intensive care unit patients as a function of age and the interaction between mechanical ventilation duration and dental treatments performed (n = 166)

Variable		n (%)	VAP		Crude RR (95CI%)	p value	Adjusted RR (95%CI)	p value
			No	Yes*				
			n (%)	n (%)				
Total		166 (100.0)	101 (60.8)	65 (39.2)	-	-	-	-
Mechanical ventilation duration (days) and dental treatment								
	≤ 14 days; without treatment	31 (18.7)	30 (96.8)	1 (3.2)	Ref.	-	Ref.	-
	≤ 14 days; with treatment	42 (25.3)	35 (83.3)	7 (16.7)	5.17 (0.67 - 39.86)	0.1152	5.33 (0.68 - 41.61)	0.1104
	> 14 days; without treatment	36 (21.7)	11 (30.6)	25 (69.4)	21.53 (3.09 - 149.84)	0.0019	21.49 (3.03 - 152.30)	0.0021
	> 14 days; with treatment	57 (34.3)	25 (43.9)	32 (56.1)	17.40 (2.50 - 121.31)	0.0039	18.13 (2.56 - 128.56)	0.0037
Mechanical ventilation duration (days) and number of dental procedures								
	≤ 14 days; ≤1 procedure	51 (30.7)	46 (90.2)	5 (9.8)	Ref.	-	Ref.	-
	≤ 14 days; > 1 procedure	22 (13.3)	19 (86.4)	3 (13.6)	1.39 (0.36 - 5.32)	0.6297	1.43 (0.37 - 5.47)	0.6056
	> 14 days; ≤ 1 procedure	71 (42.8)	27 (38.0)	44 (62.0)	6.32 (2.70 - 14.82)	< 0.0001	6.32 (2.67 - 14.94)	< 0.0001
	> 14 days; > 1 procedure	22 (13.3)	9 (40.9)	13 (59.1)	6.03 (2.45 - 14.86)	< 0.0001	6.33 (2.55 - 15.75)	< 0.0001

VAP - ventilator-associated pneumonia; RR - relative risk; 95%CI - 95% confidence interval; Ref - reference category for the independent variables.

* Outcome event.

VAP - ventilator-associated pneumonia.

## DISCUSSION

Our findings show that dental treatment did not significantly reduce the overall risk of VAP in our study population. Furthermore, survival analyses stratified by receiving dental treatment and treatment intensity did not show a clear dose-response relationship, underscoring the complexity of this association. However, interaction analyses revealed a potential protective effect within the subgroup of patients on prolonged MV (> 14 days), which should be interpreted with caution and confirmed in future studies, although similar trends have been reported elsewhere.^(7,8)^ In this study, "dental treatment" encompasses a wide range of procedures, each with potentially varying degrees of effectiveness. Furthermore, the lack of systematic oversight of the nursing staff's oral hygiene protocols may have attenuated the observed outcomes, which could be more pronounced under well-controlled conditions. Although there were concerns about chlorhexidine, its use was maintained given the high prevalence of poor oral conditions among patients in this ICU.^(9)^ Limitations include the small sample size, single-center design, limited data granularity restricting control for potential confounding factors, lack of model adjustment for oral health and disease severity, and the debated definition of VAP. Nonetheless, to our knowledge, this is the first study to apply survival analysis, offering a temporal perspective on dental treatment outcomes.

## CONCLUSION

While oral care is recognized as an important component of intensive care unit patient management, no significant association was found between dental treatment and reduction of ventilator-associated pneumonia risk in this study. Future research should address this question in larger populations, consider high-risk patient subgroups, and explore the optimal frequency of structured dental care interventions.

## Data Availability

The data cannot be made publicly available due to ethical and patient confidentiality restrictions but may be available from the authors upon reasonable request and with appropriate approvals.
