# Supplementary material for: Potential protective effect of dental treatment among subgroups of critically ill ventilated patients: a retrospective survival analysis
Source: Crit Care Sci. 2026 May 8;38:e20260305. doi: 10.62675/2965-2774.20260305 (PMC13155769; doi:10.62675/2965-2774.20260305)
Supplement: Supplementary Material [file 2965-2774-ccsci-38-e20260305-suppl01.pdf]

Critical Care Science
https://doi.org/10.62675/2965-2774.20260305

SUPPLEMENTARY MATERIAL

Potential protective effect of dental treatment among subgroups of critically ill ventilated patients: a retrospective survival analysis

Flávio de Melo Garcia[1], Caroline Tianeze de Castro[2], Renan Vicente Starling Braga[3], Josiane Celis de Almeida[3], Victor Angelo Martins Montalli[4]

EXCLUSION PROCESS

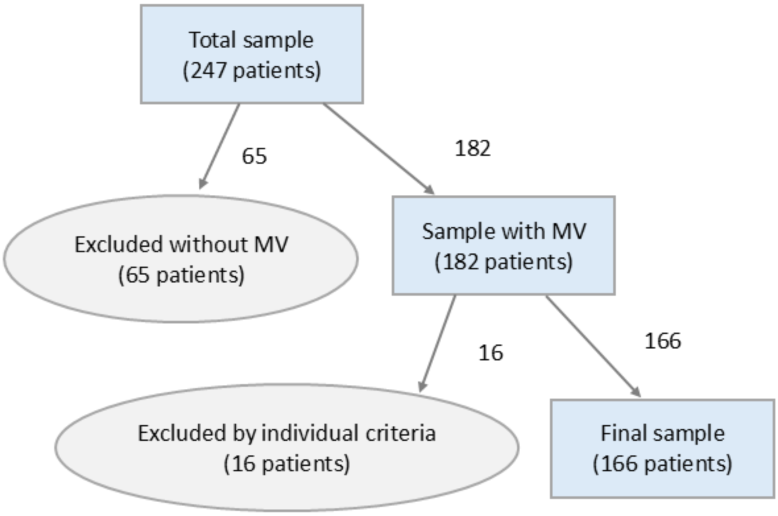

MV - mechanical ventilation.

Figure 1S - This flowchart illustrates the process of patient exclusion from the total intensive care unit sample to arrive at the final study sample of 166 patients.

Patients were primarily excluded if they did not receive mechanical ventilation or if they were excluded based on specific inclusion/exclusion criteria related to ventilator-associated pneumonia and mechanical ventilation initiation/duration.

This is an open access article under the CC BY license (https://creativecommons.org/licenses/by/4.0/).

METHODS

Dental treatment

All dental treatments throughout the study period were administered by a single designated dental professional to ensure consistency, in accordance with the established institutional protocols for intensive care unit (ICU) settings. A standardized clinical pathway was implemented for every patient, beginning with a mandatory dental evaluation conducted within 48 hours of admission. This initial assessment, guided by a standard operating procedure, facilitated the early identification of pre-existing oral health conditions and potential infectious foci.[1,2]

Following this evaluation, an individualized treatment plan was formulated and executed at the patient's bedside as soon as their clinical stability permitted. Each dental intervention systematically included a comprehensive diagnostic assessment, prophylactic debridement of visible biofilm, and the aspiration of oral and/or oropharyngeal secretions. Biofilm removal was performed mechanically using sterile gauze soaked in a 0.12% chlorhexidine gluconate solution, while secretions were managed with a dedicated suction catheter.[3,4]

When clinically indicated to control or eliminate potential sources of systemic infection, specific therapeutic procedures were performed. These included, but were not limited to, dental restorations or fissure sealants, tooth extractions, periodontal scaling, and photobiomodulation therapy. All such procedures were performed in the ICU.

In parallel with these specialist interventions, the nursing team was responsible for a separate, routine oral hygiene protocol, performed twice daily. This protocol consisted of mechanical toothbrushing and cleansing of soft tissues using gauze impregnated with 0.12% chlorhexidine, applied with a wooden tongue depressor, followed by oro-pharyngeal suctioning.[5] It is important to note, however, that adherence to this nursing-led protocol was not systematically monitored as part of this study. All data about the dental treatments, including the specific procedures performed and the total number of interventions for each patient, were meticulously extracted from the electronic medical records for analysis.

Ventilator-associated pneumonia prevention bundle

During the study period, all mechanically ventilated patients were managed according to a ventilator-associated pneumonia (VAP) prevention bundle based on the Brazilian national guidelines for healthcare-associated infections, the Institute for Healthcare Improvement (IHI) recommendations, and key evidence from the literature.[5-8]. The bundle included:

- Elevation of the head of the bed to 30 - 45°.
- Daily sedation interruption and assessment using the Richmond Agitation-Sedation Scale (RASS) to facilitate early awakening and reduce the duration of mechanical ventilation.
- Daily assessment of readiness for weaning from mechanical ventilation.
- Oral hygiene with chlorhexidine 0.12% (including toothbrushing) performed at regular intervals by nursing staff.
- Regular suctioning of oropharyngeal and tracheal secretions.
- Maintenance of endotracheal tube cuff pressure within recommended limits;
- Strict hand hygiene and standard precautions.

The use of RASS and daily awakening allows for safe reduction of sedation, facilitates patient participation in weaning trials, and contributes to decreased VAP risk by promoting earlier extubation.[9] Adherence to the bundle was monitored routinely as part of the ICU quality improvement program. This protocol served as the standard of care for all patients and was followed regardless of the dental treatment interventions performed by the ICU dental team.

Statistical analysis

Descriptive analyses were initially performed. Associations between variables were then assessed using the Chi-squared test or Fisher's Exact test, as appropriate. Subsequently, Poisson regression models with robust variance estimators were fitted to estimate the crude relative risks (RR) and 95% confidence intervals (95%CI) for VAP in relation to each independent variable. A multiple Poisson regression model with robust variance was then constructed, adjusting for age and retaining only variables with p-values ≤ 0.05 after adjustment. Adjusted relative risks (aRR) and their corresponding 95%CIs were reported. For all regression models, the reference category for each variable is explicitly indicated in the corresponding results tables (Table 1S). Model fit was evaluated using the Quasi-likelihood Information Criterion (QIC). To analyze the time between the initiation of MV and the onset of VAP, survival analysis techniques were applied. The Kaplan–Meier method was used to estimate the probability of developing VAP over time. In this context, VAP was considered a failure event, while discharge or death without VAP was treated as censoring. Survival probabilities and curves were stratified by dental treatment implementation. The Cox proportional

hazards model was used to estimate the hazard of developing VAP, with results expressed as hazard ratios (HRs) and 95%CIs. Stratified Cox models were also constructed for the variables "dental treatment provided" (none *versus* yes) and "number of treatments" (≤ 1 *versus* > 1). The Wald test was applied to assess the statistical significance of the HRs. All analyses were conducted in R (R Core Team, 2025) with a significance level of $\alpha = 0.05$.[10]

RESULTS OF THE BIVARIATE ANALYSIS

Table 1S - Frequency of discharge, development of ventilator-associated pneumonia, and deaths after the initiation of mechanical ventilation among intensive care unit patients who did or did not receive dental treatment (n = 166)

Outcomes	Without dental treatment n (%)	With dental treatment n (%)	Total n (%)
No VAP and discharged	16 (23.9)	22 (22.2)	38 (22.9)
VAP and discharged	12 (17.9)	18 (18.2)	30 (18.1)
No VAP and death	25 (37.3)	38 (38.4)	63 (38.0)
VAP and death	14 (20.9)	21 (21.2)	35 (21.1)
Total	67 (100.0)	99 (100.0)	166 (100.0)

VAP - ventilator-associated pneumonia. p = 0.9958 (Chi-squared test)

Table 2S - Frequency of discharge, development of ventilator-associated pneumonia, and deaths after the initiation of mechanical ventilation among intensive care unit patients who underwent one or more dental procedures (n = 166)

Outcomes	Up to 1 procedure n (%)	More than 1 procedure n (%)	Total n (%)
No VAP and discharged	30 (24.6)	8 (18.2)	38 (22.9)
VAP and discharged	21 (17.2)	9 (20.5)	30 (18.1)
No VAP and death	43 (35.2)	20 (45.5)	63 (38.0)
VAP and death	28 (23.0)	7 (15.9)	35 (21.1)
Total	122 (100.0)	44 (100.0)	166 (100.0)

VAP - ventilator-associated pneumonia. p = 0.4905 (Chi-squared test)

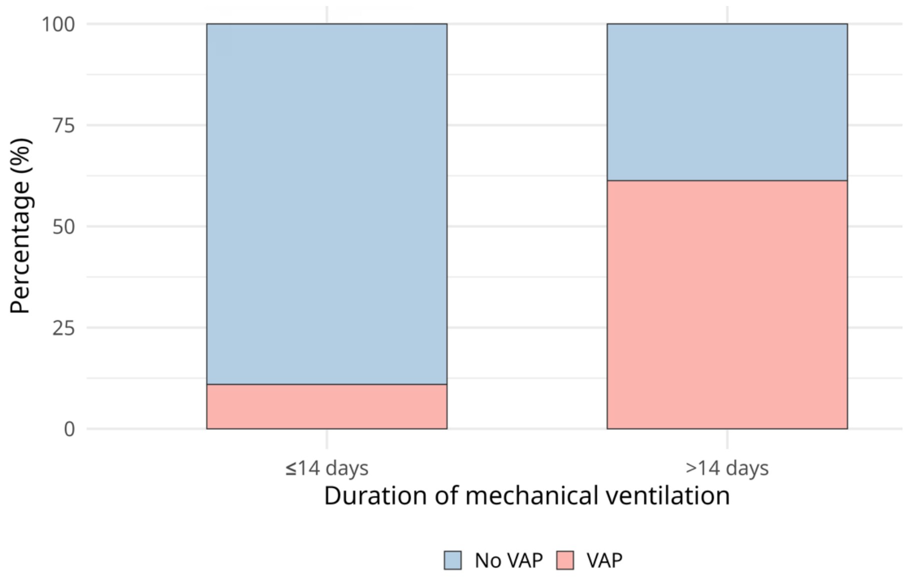

VAP - ventilator-associated pneumonia.

Figure 2S - Ventilator-associated pneumonia in intensive care unit patients according to duration of mechanical ventilation (n = 166).

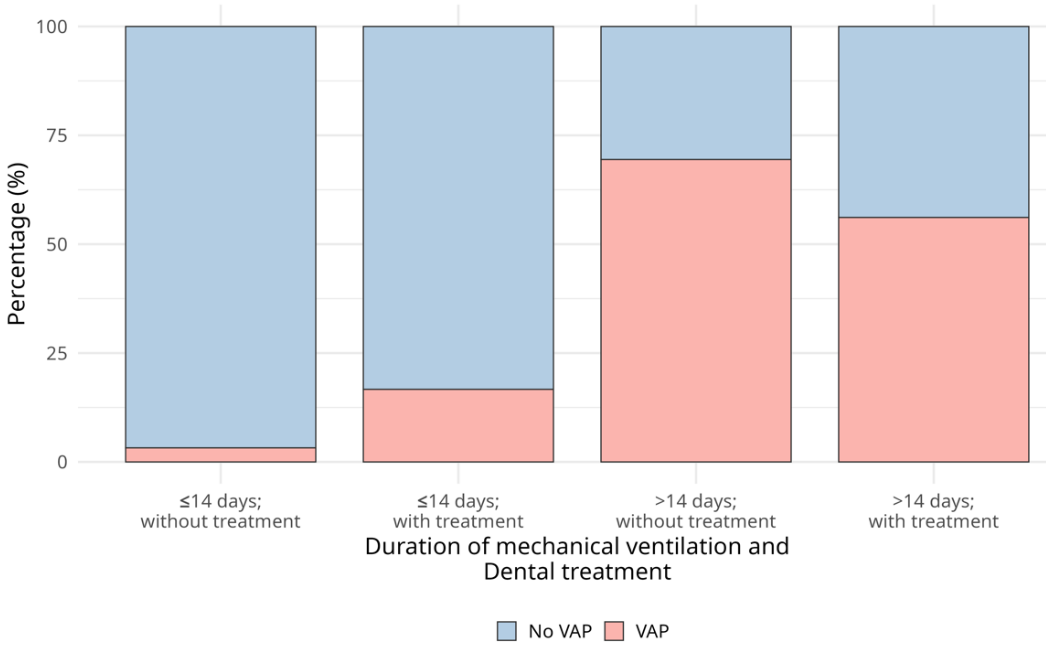

VAP - ventilator-associated pneumonia.

Figure 3S - Ventilator-associated pneumonia in intensive care unit patients according to mechanical ventilation duration and dental treatment (n=166).

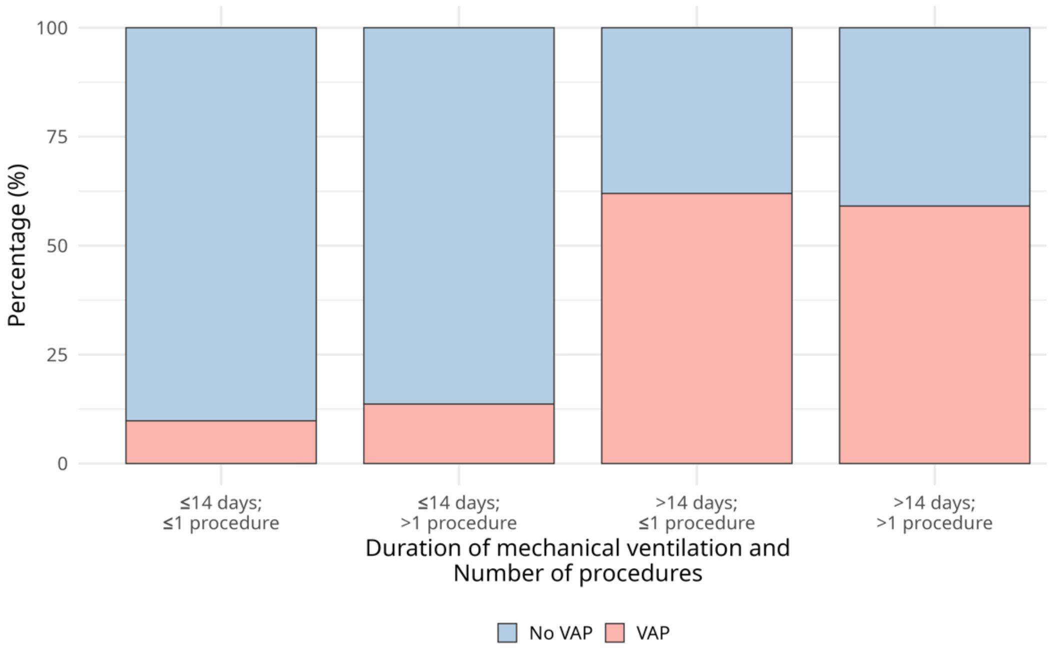

VAP - ventilator-associated pneumonia.

Figure 4S - Ventilator-associated pneumonia in intensive care unit patients according to mechanical ventilation duration and the number of dental procedures performed (n = 166).

COMPLEMENTARY RESULTS OF THE SURVIVAL ANALYSIS

Table 3S - Estimated probability of developing ventilator-associated pneumonia and 95% confidence interval over the duration of mechanical ventilation, by group (n = 166)

Duration of MV (days)	Dental treatment		Number of dental procedures	
	Without	With	≤ 1	> 1
5	0.037 (0.000 - 0.086)	0.103 (0.037 - 0.164)	0.065 (0.017 - 0.110)	0.109 (0.002 - 0.204)
10	0.213 (0.076 - 0.330)	0.266 (0.156 - 0.361)	0.218 (0.124 - 0.303)	0.321 (0.130 - 0.471)
15	0.467 (0.268 - 0.612)	0.359 (0.230 - 0.467)	0.371 (0.249 - 0.473)	0.478 (0.231 - 0.646)
20	0.612 (0.370 - 0.760)	0.559 (0.392 - 0.680)	0.568 (0.412 - 0.682)	0.608 (0.316 - 0.776)
25	0.792 (0.499 - 0.914)	0.694 (0.470 - 0.823)	0.769 (0.566 - 0.877)	0.608 (0.316 - 0.776)
30	0.931 (0.569 - 0.989)	0.885 (0.434 - 0.977)	0.931 (0.627 - 0.987)	0.804 (0.128 - 0.956)

MV - mechanical ventilation.

Table 4S - Probability and confidence interval of developing ventilator-associated pneumonia according to dental treatment (n = 166)

Category	Hazard ratio (95%CI)	p value
Dental treatment		
Without	1.20 (0.72 - 2.,00)	0.4827
With	Ref	-
Number of dental procedures		
≤ 1	1.00 (0.56 - 1.77)	0.9876
> 1	Ref	-

95%CI - 95% confidence interval.

ADDITIONAL RESULTS

Table 5S - Descriptive analysis of oral conditions identified during clinical examination and treatments performed in intensive care unit patients (n = 166)

Variables	n (%)
Tongue coating	61 (36.7)
Periodontal disease*	77 (46.7)
Edentulism	24 (14.5)
Untreated dental lesions†	28 (16.9)
Oral mucosal lesions	40 (24.1)
Photobiomodulation	44 (26.5)
Tooth extraction	31 (18.7)
Scaling	46 (27.7)
Restorative treatment	11 (6.6)
Others procedures	20 (12.0)
Total	166 (100.0)

* Includes both gingivitis and periodontitis; † includes both cavitated carious lesions and retained roots.

REFERENCES

1. Pains MB, de Melo NS, Leite AF, Mesquita CR, de Souza Figueiredo PT. Contribution of dental treatment to reducing mortality in the ICUs: a 6-year retrospective analysis. Sci Rep. 2025;15(1):18188.
2. da Cruz MK, Morais TM, Trevisani DM. Clinical assessment of the oral cavity of patients hospitalized in an intensive care unit of an emergency hospital. Rev Bras Ter Intensiva. 2014;26(4):379-83.
3. Huang H, Yu X, Huang C, Zeng J, Li Y. Oral care medications for the prevention and treatment of ventilator-associated pneumonia in intensive care unit. Front Oral Health. 2025;6:1566355.
4. Winning L, Lundy FT, Blackwood B, McAuley DF, El Karim I. Oral health care for the critically ill: a narrative review. Crit Care. 2021;25(1):353.
5. Institute for Healthcare Improvement. How-to Guide: Prevent Ventilator-Associated Pneumonia. Cambridge, MA: Institute for Healthcare Improvement; 2012. [cited 2025 Oct]. Available at: http://www.ihi.org
6. Blot S, Ruppé E, Harbarth S, Asehnoune K, Poulakou G, Luyt CE, et al. Healthcare-associated infections in adult intensive care unit patients: changes in epidemiology, diagnosis, prevention and contributions of new technologies. Intensive Crit Care Nurs. 2022;70:103227.
7. Papazian L, Klompas M, Luyt CE. Ventilator-associated pneumonia in adults: a narrative review. Intensive Care Med. 2020;46(5):888-906.
8. He Q, Wang W, Zhu S, Wang M, Kang Y, Zhang R, et al. The epidemiology and clinical outcomes of ventilator-associated events among 20,769 mechanically ventilated patients at intensive care units: an observational study. Crit Care. 2021;25(1):44.
9. Li W, Cai J, Ding L, Chen Y, Wang X, Xu H. Incidence and risk factors of ventilator-associated pneumonia in the intensive care unit: a systematic review and meta-analysis. J Thorac Dis. 2024;16(9):5518-28.
10. R Core Team. R: A language and environment for statistical computing. Vienna, Austria: R Foundation for Statistical Computing; 2025 [cited 2025 Jul 12]. Available from: https://www.R-project.org/
